# Comparative Study of Electrochromic Supercapacitor Electrodes Based on PEDOT:PSS/ITO Fabricated via Spray and Electrospray Methods

**DOI:** 10.1021/acsomega.4c04235

**Published:** 2024-06-28

**Authors:** Fahri Çatoğlu, Sinem Altınışık, Sermet Koyuncu

**Affiliations:** † Department of Chemical Engineering, Canakkale Onsekiz Mart University, 17100 Canakkale, Türkiye; ‡ Department of Energy Resources and Management, Canakkale Onsekiz Mart University, 17100 Canakkale, Türkiye

## Abstract

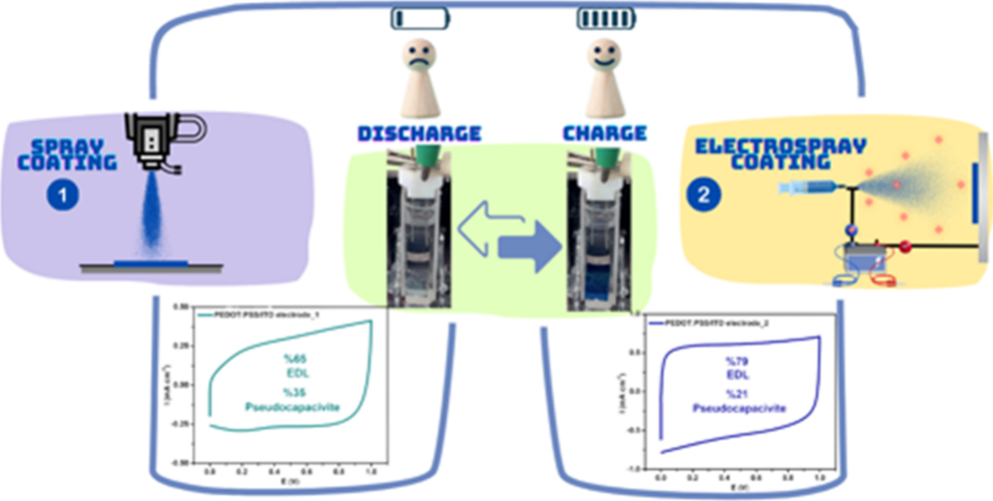

PEDOT:PSS stands out as a leading commercial conducting polymer due to its excellent water dispersibility, controllable miscibility, adjustable conductivity, and ability to form films through various techniques. This study investigates the electrochemical and electrochromic performance of electrodes prepared by depositing PEDOT:PSS onto ITO surfaces by using two distinct methods: conventional spray coating and electrospray deposition. Detailed characterization of the prepared electrodes was performed by using atomic force microscopy, scanning electron microscopy, Fourier-transform infrared, and Raman spectroscopy techniques. Our findings reveal that electrodes fabricated via electrospray deposition (PEDOT:PSS/ITO electrode_2) significantly outperform those made by spray coating (PEDOT:PSS/ITO electrode_1). Specifically, electrode_2 exhibits a capacitance of 1678.60 μF cm^−2^, compared to 826.14 μF cm^−2^ for electrode_1, at a current density of 10 μA cm^−2^. PEDOT:PSS electrodes exhibit areal energy densities of 0.41 and 0.84 mW h cm^−2^, along with power densities of 4.96 and 4.97 μW cm^−2^, respectively. Moreover, electrode_2 demonstrates a high coloration efficiency of 84.32 cm^2^ C^−1^ and fast response times of 1.36 s for coloration and 0.98 s for bleaching. This study highlights the advantages of electrospray deposition over traditional methods, showcasing the potential of electrospray-prepared PEDOT:PSS electrodes for use in multifunctional energy storage devices.

## 1 Introduction

The need to transition from traditional energy sources to renewable alternatives has spurred significant interest in the development of efficient energy storage devices.^1,2^ In recent years, supercapacitors (SCs) have garnered substantial interest within the realm of energy storage systems, attributed to their rapid charge−discharge capabilities, impressive power density, and exceptional cycle stability.^3^ Another important topic, electrochromism refers to the reversible alteration of color induced by an applied voltage, a phenomenon driven by electrochemical redox reactions occurring within the electrochromic (EC) materials.^4−6^ The charge injection and extraction occurring during the charge−discharge phases of the SC align with the chromatic transitions observed at various potentials, providing an effective indication of energy storage levels.^7^ Combining EC features with SCs and crafting self-charging power packages equipped offer improved user convenience and functionality.^8,9^ Among these, enabling individuals to easily and accurately predict or determine the status of electrical energy storage in real-time is essential.^10−12^ Therefore, the utilization or integration of smart materials that facilitate the easy determination of electrical energy storage holds significant importance.

Over the past decades, conductive polymers (CPs) have garnered increasing attention due to their promising potential to supplant their inorganic counterparts. Typically characterized by alternating single and double bonds, CPs possess π-conjugated systems that underlie their unique optical, electrochemical, and electrical/electronic properties.^13,14^ The combined ionic/electronic mixed conductivity of CPs has generated particular interest in supercapacitor applications, particularly for facilitating sensitive charge transfer at the interface with an ionically conductive medium.^15^ Among current pseudocapacitive materials, CPs exhibit superior electrical conductivity compared to transition metal oxides, positioning them as promising electrode materials for SCs.^16,17^ Moreover, coating metal-based electrodes with CPs presents a significant advantage: by capitalizing on CPs’ volumetric charge-transfer capacity, it enhances the electrode capacitance at the interface, thereby reducing impedance. Poly(3,4-ethylenedioxythiophene) (PEDOT), recognized for its environmentally friendly, stable, and easily processable nature along with its electrical conductivity and electrochemical performance, is synergistically combined with polystyrenesulfonate (PSS) to augment solubility.^18,19^ This incorporation, denoted as PEDOT:PSS, enhances solution processability and finds extensive application as a hole-transporting material (HTM) in electronic devices, owing to its outstanding properties such as high transparency, flexibility, and cost-effectiveness. PEDOT:PSS offers the advantage of being prepared via a variety of coating techniques, including spin coating, spray coating, electrodeposition, electrospinning, and electrospray deposited, thus increasing its versatility in electronic materials.^20,21^ Modifying the thickness of PEDOT:PSS coatings significantly impacts the electrode stability. Investigating the influence of PEDOT:PSS coating thickness reveals a notable trend: as the thickness increases, the electrochemical impedance of the electrode decreases, concurrently leading to an increase in the charge storage capacity.^22,23^ This correlation underscores the pivotal role of coating thickness in fine-tuning the electrochemical properties of the electrode, thereby offering valuable insights for optimizing its performance in diverse applications such as sensors.^24−27^

One of the studies focusing on EC-SC hybrid systems utilizing PEDOT:PSS presents a hybrid film composed of self-assembled silver nanoparticles exhibiting high conductivity, transparency, oxidation stability, and flexibility, along with a periodic, uniform grid of silver sintered on a poly(ethylene terephthalate) (PET) substrate.^28^ The hybrid film was reported to retain an optical modulation of 87.7% and a specific capacitance of 67.2% at 10 A/g^−1^, showing remarkable performance compared to the initial values observed at 1 A/g^−1^. In another study, Yun et al. developed the EC-SC system consisting of Au/Ag core−shell nanowire-embedded polydimethylsiloxane (PDMS) and double-sided WO_3_ nanotube/PEDOT:PSS. They stated that the incorporation of a PEDOT:PSS wrapping layer onto the WO_3_ nanotube electrode improved coloration efficiency by 20.4% to 83.9 cm^2^ C^−1^ and specific capacity by 38.6% to 471.0 F g^−1^, leading to increased energy and power densities with maximum values of 19.1 kW kg^−1^ and 52.6 Wh kg^−1^, respectively.^29^ Eisenberg et al. developed an EC-SC hybrid system consisting of a coordination-based network of metal complexes bound to fluorine-doped tin oxide (FTO)-coated glass as the battery-like electrode and a combination of multiwalled carbon nanotubes (MWCNTs) deposited on a layer of PEDOT:PSS directly attached to FTO as the capacitive-like electrode.^30^ The device is noted to operate effectively in a low potential range of −0.6 to 2 V, exhibiting notable energy and power densities of approximately 2.2 Wh kg^−1^ and 2529 W kg^−1^, respectively. It has also been shown to exhibit a high Coulomb efficiency of 99% with a short charging time of approximately 2 s and maintain a charge retention time (V1/2) of approximately 60 min. They have proven their remarkable stability in both color and energy over more than 1000 consecutive charge−discharge cycles. In addition, Moniz et al. successfully demonstrated that electrospray deposition of PEDOT:PSS on carbon yarn electrodes significantly enhances the performance of solid-state flexible supercapacitors. Their results showed a high specific capacitance of 72 mF g^−1^ and a cyclic stability of more than 85% capacitance retention after 1500 cycles.^31^

**Scheme 1:**
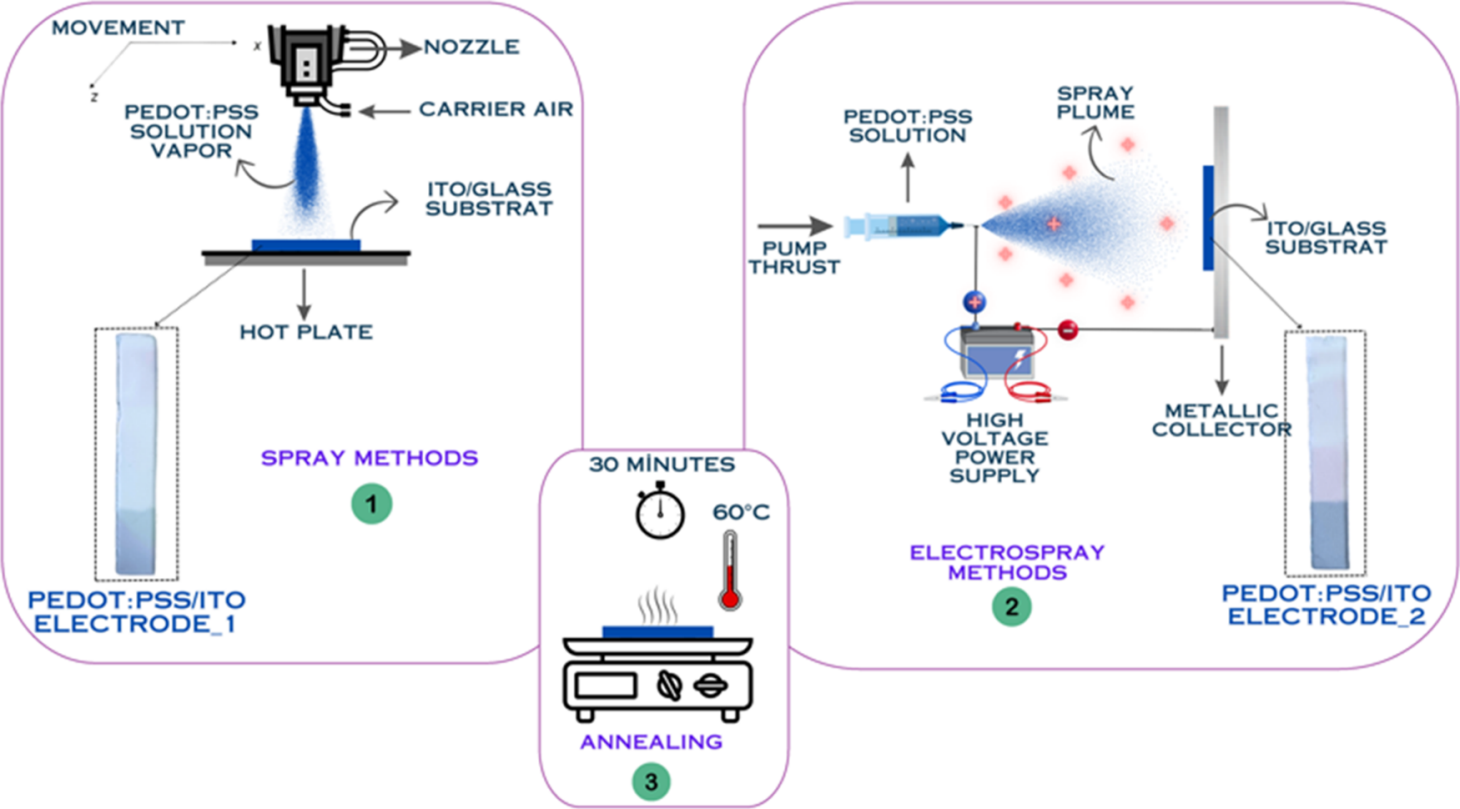
Preparation Procedure of PEDOT:PSS/ITO Electrode_1 and PEDOT:PSS/ITO Electrode_2

Herein, we successfully fabricated EC-SC electrodes by simple methods using an ITO/glass, thereby integrating electronic and optical properties into thin films using pristine PEDOT:PSS, providing a simplified and cost-effective solution (see Supporting Information). Our primary focus lies in the advancement of EC-SC thin films, accomplished solely through the application of pristine PEDOT:PSS coating onto the ITO/glass surface. Moreover, a comparative analysis of films prepared using two different coating techniques was conducted to assess the capacitance and electrochromic properties of the electrodes based on their surface morphology (Scheme 1 and Figure S1a,b). Remarkably, the EC-SC electrode generated using the electrospray method showcased a capacitive effect (1678.60 μF cm^−2^ at 10 μA cm^−2^) along with a high coloration efficiency (84.32 cm^2^ C^−1^) and outstanding electrochromic performance. These results underscore the potential applications of these electrodes in smart windows, where they can seamlessly integrate energy storage capabilities with electrochromic functionalities.

## 2 Experimental Section

### 2.1 Fabrication of PEDOT:PSS/ITO Electrode

The commercial ITO/glass was initially subjected to ultrasonication cleaning with acetone and methanol for 20 min each, followed by drying at 90 °C for 10 min. Subsequently, the substrates underwent UV/ozone treatment for 10 min. Before the coating procedures, the PEDOT:PSS solution (CLEVIOS PH 500) was diluted with a mixture of IPA/EG/(PEDOT:PSS) = 2:1:5 (vol %) and stirred for over an hour. Subsequently, the solution was filtered through a 0.45 mm filter.

In the fabrication process of thin films via spray coating, the equipment comprises a 50 mL reservoir, a compressor for delivering carrier airflow to disperse spray droplets, an atomizing nozzle, and a heated plate for controlling the substrate temperature (Figure S1a) (set at 60 °C for this study). A 3D atomizing nozzle system, actuated by a motor, was employed to ensure precise control over spray parameters, operating at a designated speed and strategy (utilizing four spray passes). Prior to nozzle entry, a pressure-regulating valve adjusts the carrier air pressure to 0.4 MPa. The nozzle-to-substrate distance was maintained at a constant 10 cm, while the lateral nozzle velocity was set to 120 mm s^−1^. Subsequently, the fabricated spray films underwent annealing at 60 °C for a duration of 30 min.

The electrospray coating system consists of a 5 mL syringe connected to a needle with a metallic tip measuring 0.45 mm × 13 mm, a syringe pump, and a fixed plate where the coating will accumulate. In this setup, the needle and syringe are horizontally aligned with the substrate (Figure S1b). The electrospray coating parameters have been optimized to have a flow rate of 60 μL h^−1^, an applied voltage of 18 kV, a needle tip-to-collector distance of 15 cm, and a coating duration of 30 min. The coating process was maintained at a constant room temperature (18−25 °C) and relative humidity ranging between 40 and 44%. The films prepared postcoating were annealed at 60 °C. Consequently, uniform PEDOT:PSS-based electrodes with approximately 500 nm thickness are obtained.

### 2.2 Characterizations

The Fourier-transform infrared spectroscopy (FT-IR) spectra were acquired using an ATR system on a Cary 630 FT-IR Spectrometer (Agilent Technologies), covering the spectral range from 2000 to 650 cm^−1^. Raman spectra excited by means of a visible diode laser (532 nm) were collected on a WITEC ALPHA 300RA Raman microspectrometer. Atomic force microscopy (AFM, Nanosurf Naio) and scanning electron microscopy (SEM, JEOL JSM-7100-F) were employed to characterize the surface morphologies of the PEDOT:PSS/ITO electrodes. Electrochemical measurements were performed by using a BioLogic SP-50e electrochemical workstation. The transmittance spectra of electrodes and device were tested using a UV−visible spectrophotometer (Analytic Jena Speedcord S600 diode-array). The spectroelectrochemical cell consists of an Ag wire (RE), a Pt wire (CE), and an ITO/electrode as the transparent working electrode in a quartz cell (supporting electrolyte; 0.1 M LiClO_4_, solvent; ACN).

## 3 Results and Discussion

### 3.1 PEDOT:PSS-Based Electrode Preparation and Characterization

Here, we develop an effective fabrication strategy for pristine PEDOT:PSS-based EC-SC electrodes using two different methods. Prior to coating, the PEDOT:PSS solution is diluted with a mixture of IPA/EG/(PEDOT:PSS) = 2:1:5 (vol %) and filtered. As shown in Scheme 1, for electrospray coating, a system is set up with a syringe connected to a needle of specific dimensions, a syringe pump, and a fixed plate. Parameters are optimized: flow rate at 60 μL h^−1^, voltage at 18 kV, needle tip-to-collector distance at 15 cm, and coating duration of 30 min. For spray coating, parameters are set as follows: substrate temperature of 60 °C, carrier air pressure of 0.4 MPa, nozzle-to-substrate distance of 10 cm, and lateral spray velocity of 120 mm s^−1^. Fabricated films undergo annealing at 60 °C for 30 min. Consequently, the uniform PEDOT:PSS-based electrodes with a thickness of approximately 500 nm were obtained, determined by AFM profilometer results taken by scratching the surfaces (Figure S2). The chemical structures of PEDOT:PSS-based electrodes were examined by Raman and FT-IR techniques (Figures S3 and S4). The Raman spectrum of PEDOT:PSS reveals two weak bands at 1557 cm^−1^ (quinoid structure) and 1504 cm^−1^ (C_α’_ =C_β’_ stretching), alongside a strong band at 1431 cm^−1^ (symmetric C_α_=C_β_ stretching vibrations), and one at 1362 cm^−1^ (C_β_–C_β_ stretching). Additional peaks are observed at 1258 cm^−1^ (C_α_–C_α’_ inter-ring stretching). The band at 1431 cm^−1^ is particularly significant as it reflects the oxidation (doping) level of PEDOT:PSS, which, due to the more porous structure of the electrode coated by the electrospray method, is observed at approximately 1438 cm^−1^ with a shift of about 7 cm^−1^.^32^ This shift may also be attributed to a decrease in the ratio of PSS counterion structures within the PEDOT chain depending on the coating method.^33^ Furthermore, in the FT-IR spectrum of PEDOT:PSS electrodes, the peaks at 1599 and 1520 cm^−1^ correspond to the C=C stretching in the aromatic rings of PSS and the thiophene ring of PEDOT, respectively. The peak at 1412 cm^−1^ indicates the C−C stretching in the thiophene ring of PEDOT. The symmetric and asymmetric stretching of S=O can be observed at 1167 and 1040 cm^−1^, respectively, attributed to PSS and the oxidant SO_4_^−2^. The triple peaks at 917, 840, and 676 cm^−1^ correspond to the C−S stretching in the thiophene ring of PEDOT.^34^ Additionally, the broadening of the FT-IR peaks for PEDOT:PSS/ITO electrode_2 is attributed to the rougher surface texture of the electrode.

**Figure 1:**
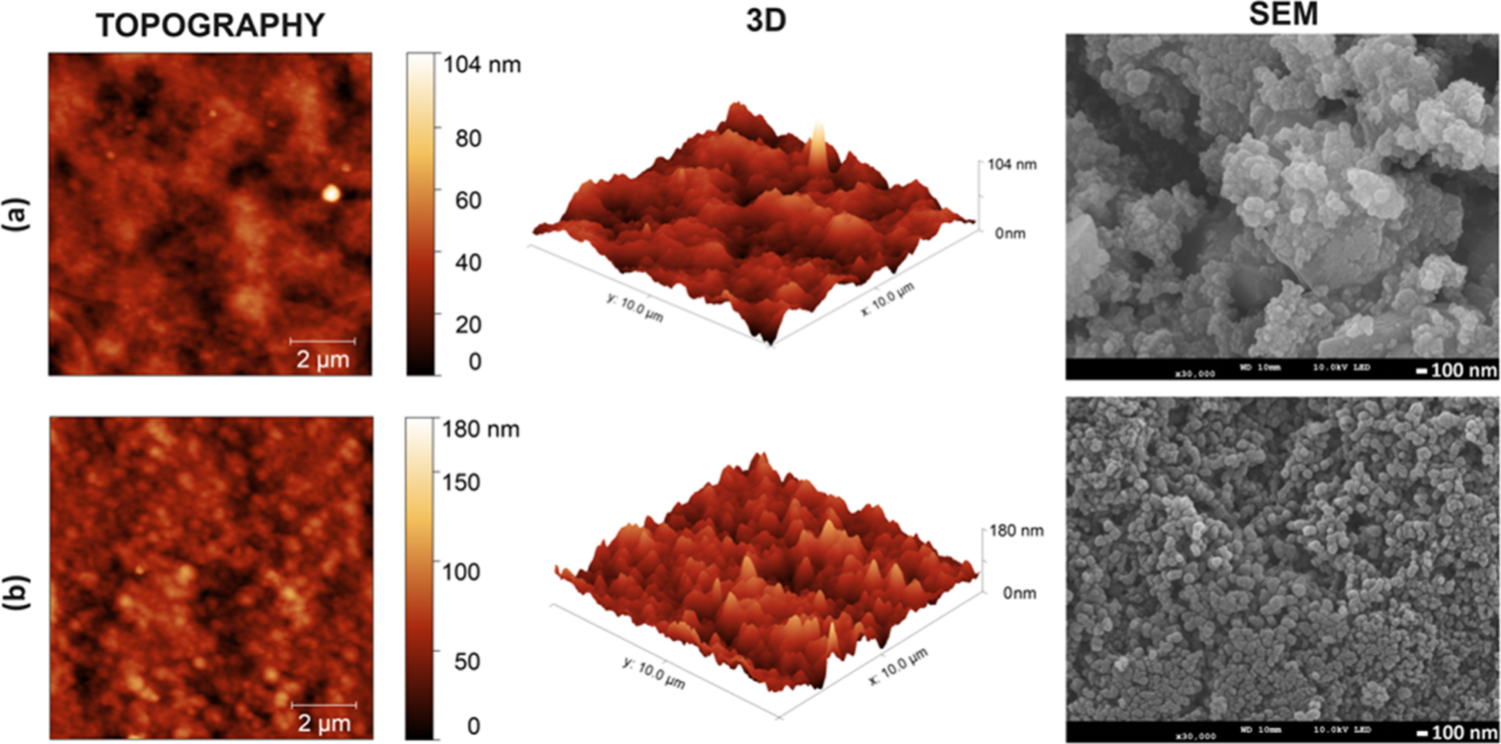
AFM and field emission SEM (FESEM) images of PEDOT:PSS/ITO electrode_1 (a) and PEDOT:PSS/ITO electrode_2 (b).

The AFM and SEM images indicate a clear change in the morphology of films depending on the different coating methods (Figure 1a,b). According to the topographic image, larger grains with weak phase separation between PEDOT and PSS chains are observed in PEDOT:PSS/ITO electrode_1 prepared by the spray method. In addition, in PEDOT:PSS/ITO electrode_2 prepared by the electrospray method, a better phase separation between PEDOT and PSS chains is observed due to the electrical alignment effect of the applied process, resulting in smaller domains and a more homogeneous separation. These particles in the range of approximately 100−200 nm may increase the interaction with the electrolyte by providing a wide surface area on the electrode surface. These grain boundaries between domains act as transport barriers for charge carriers during charge transport, making them crucial for the hopping of charge carriers. Furthermore, the root mean surface (RMS) roughness of PEDOT:PSS/ITO electrode_1 and PEDOT:PSS/ITO electrode_2 is 2.19 and 6.72 nm, respectively. A sufficiently, large and homogeneous distribution of grains eliminates hopping as the limiting factor via phase boundaries.^35,36^ The spherical shape of PEDOT:PSS particles can be attributed to the formation of spherical grains by PSS in the SEM images. Particle clusters were determined as aggregates in PEDOT:PSS/ITO electrode_1, whereas SEM images also corroborated that particles in PEDOT:PSS/ITO electrode_2 were distributed quite uniformly. Thus, the morphology observed in PEDOT:PSS/ITO electrode_2, influenced by the distribution ratio of PEDOT to PSS and nanograin packing density, emerges as a better candidate in EC-SC applications.

### 3.2 Supercapacitive Properties of PEDOT:PSS/ITO Electrodes

**Figure 2:**
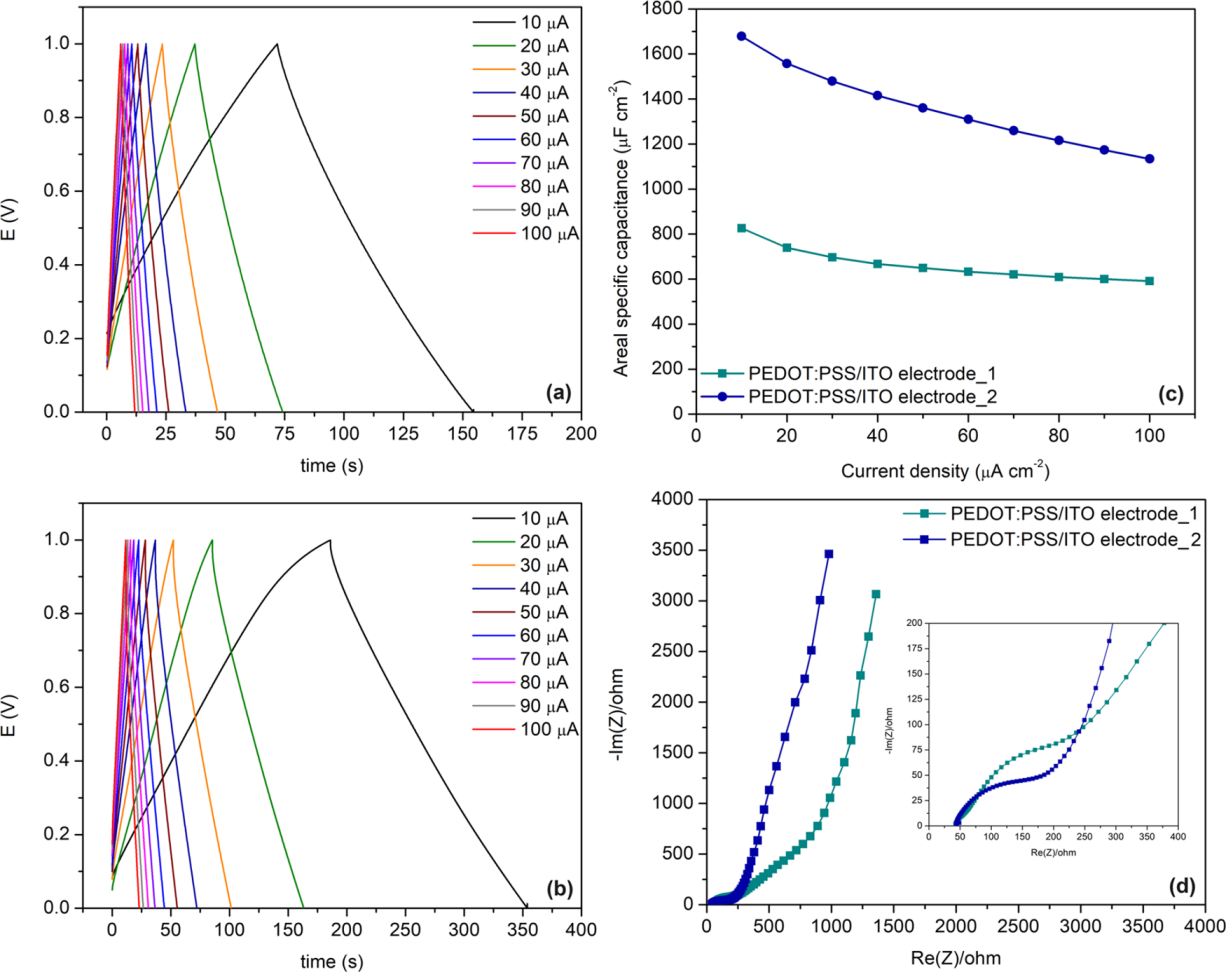
Galvanostatic charge and discharge curves of PEDOT:PSS/ITO electrode_1 (a) and PEDOT:PSS/ITO electrode_2 (b). Capacitance values of PEDOT:PSS/ITO electrode_1 and PEDOT:PSS/ITO electrode_2 at various current densities are shown in (c). Nyquist curves of PEDOT:PSS/ITO electrode_1 and PEDOT:PSS/ITO electrode_2 electrodes with doping modes (d).

The capacitive performance stands as the pivotal criterion in determining the suitability of polymers for the fabrication of the EC-SC application. The supercapacitor performance of PEDOT:PSS/ITO electrodes prepared via spray and electrospray coating methods was evaluated in ACN/LiClO_4_ solution at different current densities through galvanostatic charge−discharge (GCD) measurements within the potential range of 0.0–1.0 V. The charge−discharge curves of PEDOT:PSS/ITO electrodes with good symmetry exhibited an ideal triangular shape, indicating typical capacitance characteristics of the electrodes and fast charge−discharge capability (Figure 2a,b). The areal-specific capacitances of PEDOT:PSS/ITO electrode_1 and PEDOT:PSS/ITO electrode_2 electrodes were calculated to be 826.14 and 1678.60 μF cm^−2^, respectively, at a current density of 10 μA cm^−2^. These specific capacitance values are reasonable when considered for a pristine electrode system.^37−41^ The relationship between areal-specific capacitance and current density, as shown in Figure 2c, indicates that the areal-specific capacitance of PEDOT:PSS/ITO electrodes decreases with increasing current density. At 10 μA cm^−2^ current density, PEDOT:PSS/ITO electrode_1 showcases an areal-specific capacitance of 826.2 μF cm^−2^, whereas at 100 μA cm^−2^, it retains 71.6% of its initial capacitance (591.2 μF cm^−2^). As for PEDOT:PSS/ITO electrode_2, at a 10 μA cm^−2^ current density, it exhibits an areal-specific capacitance of 1678.6 μF cm^−2^, declining to 67.6% of its initial capacitance (1133.9 μF cm^−2^) at 100 μA cm^−2^. PEDOT:PSS electrodes also exhibit areal energy densities of 0.41 and 0.84 mW h cm^−2^, along with power densities of 4.96 and 4.97 μW cm^−2^, respectively. Furthermore, the enhanced capacitance of PEDOT:PSS/ITO electrode_2, calculated at a current density of 10 μA cm^−2^, can be explained by two main factors: First, the porous structure of PEDOT:PSS/ITO electrode_2 enhances the specific surface area of the polymer film; Second, the PEDOT:PSS/ITO electrode_2 displays a diminished charge-transfer resistance (*R*_ct_) (Figures 2d and 2d inset), thereby facilitating rapid ion diffusion rates. These findings underscore the favorable capacitance and rapid charging attributes of PEDOT:PSS/ITO electrode_2. Taking into account all of these findings, the results obtained for the pristine PEDOT:PSS electrode are comparable to those reported for other doped PEDOT:PSS electrodes in the literature (Table S1).

**Figure 3:**
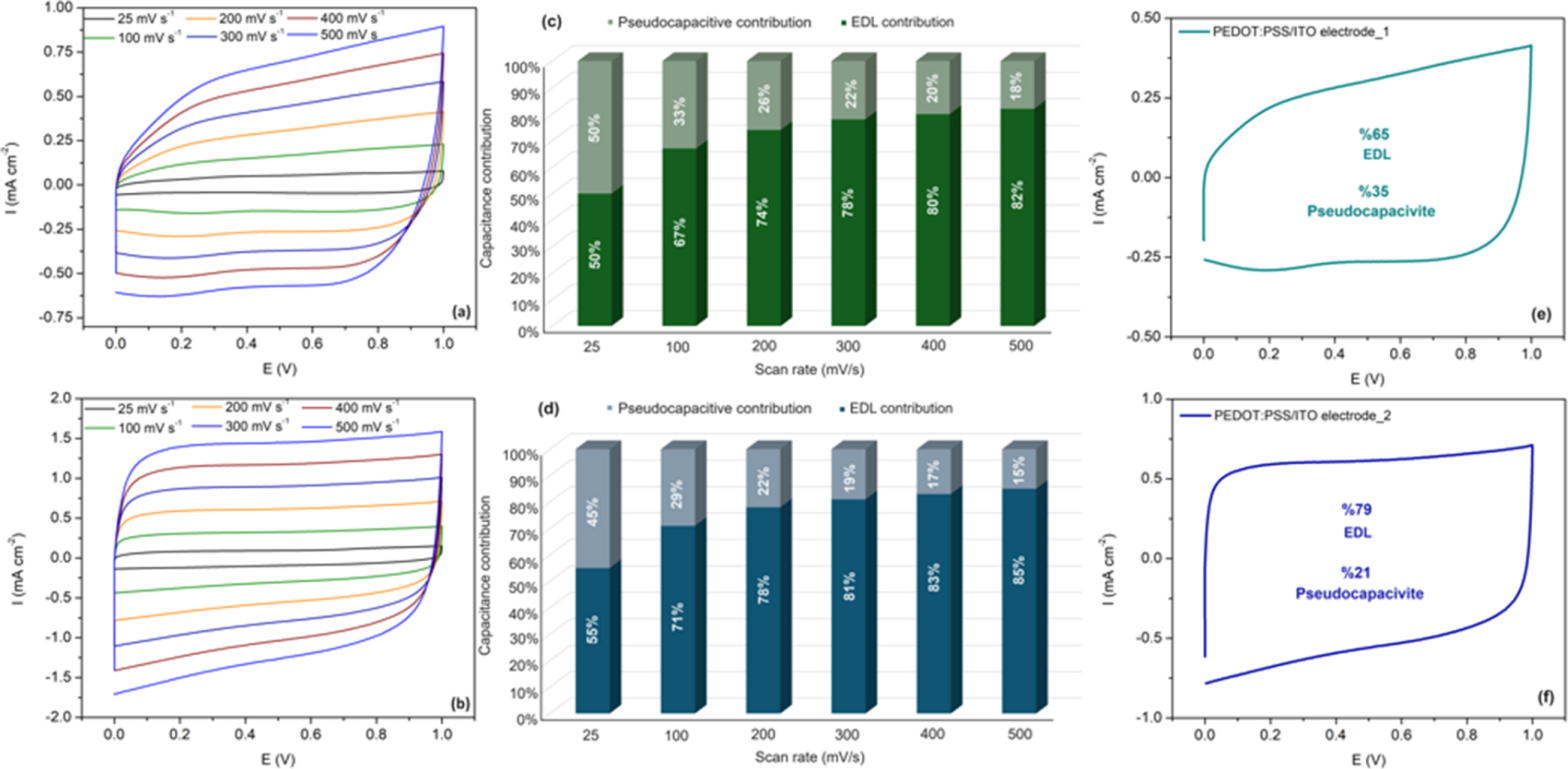
CV curves of PEDOT:PSS/ITO electrode_1 (a) and PEDOT:PSS/ITO electrode_2 (b). Percentage of EDL and pseudocapacitive behavior at different scanning rates for PEDOT:PSS/ITO electrode_1 (c) and PEDOT:PSS/ITO electrode_2 (d). Dunn’s method analysis of the capacitance contribution of PEDOT:PSS/ITO electrode_1 (e) and PEDOT:PSS/ITO electrode_2 (f) at 200 mV s^−1^.

Electrical double layer (EDL) and pseudocapacitive controlled processes are determined by analyzing the CV data according to Dunn equation (*i* = *k*_1_*ν* + *k*_2_*ν*^0.5^), where the first term, *k*_1_*ν*, represents the current contributed by the EDL effect, while the second term, *k*_2_*ν*^0.5^, accounts for the current associated with pseudocapacitive reactions.^42,43^ In addition, the sweep rate dependence of the current is shown for constant potentials between 0.0 and 0.9 V in Figure S5. Although the capacitive mechanism, activated by the electrostatic force on the surface for charge storage, is dominant for both electrodes, it is especially more pronounced on PEDOT:PSS/ITO electrode_2 (Figure 3a,b). The contributions of EDL and pseudocapacitive to the total capacity were calculated at 200 mV s^−1^ as 65 and 35% for PEDOT:PSS/ITO electrode_1 and 79 and 21% for PEDOT:PSS/ITO electrode_2, respectively (Figure 3e,f). Additionally, the scan-rate-dependent capacitive contribution is summarized for both electrodes at 0.5 V (Figure 3c,d). These results showed that the EDL effect was more dominant than the pseudocapacitive effect during ion doping and dedoping of PEDOT:PSS at increasing scanning rate. Herein, the capacitive property of PEDOT:PSS electrodes is associated with two redox peaks, indicating the intercalation/adsorption of both anions (ClO_4_^−^) and cations (Li^+^) during electrochemical switching.^44^

### 3.3 Electrochromic Properties of PEDOT:PSS/ITO Electrodes

**Figure 4:**
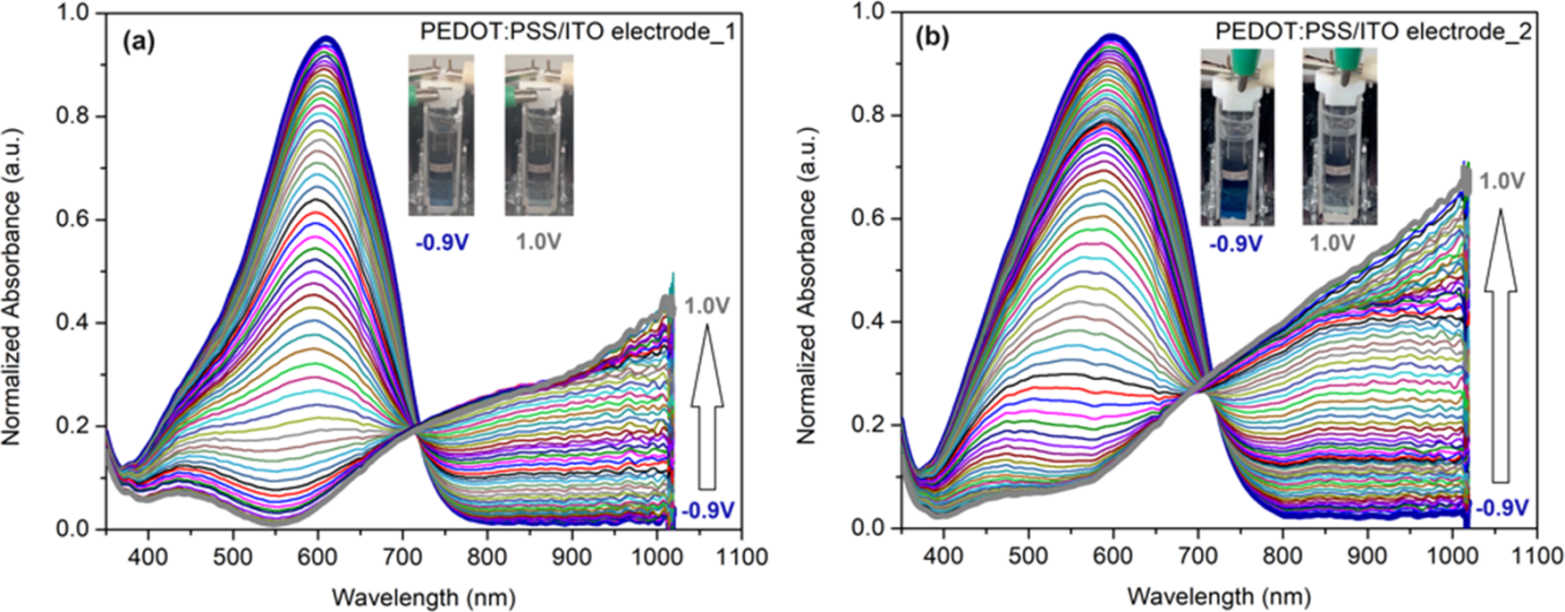
Spectroelectrochemical properties of PEDOT:PSS/ITO electrode_1 (a) and PEDOT:PSS/ITO electrode_2 (b).

The spectroelectrochemical properties of PEDOT:PSS-based electrodes prepared by spray and electrospray methods were compared in LiClO_4_(0.1 M)/ACN (Figure 4a,b). Neutral films exhibit a broad band centered at about 590 nm corresponding to a dark blue color at −0.9 V. During the oxidation process, a clean and homogeneous transition takes place within the potential range of −0.9 to 1.0 V. This transition results in the disappearance of the 590 nm band and increased absorption in the NIR region, with an isosbestic point observed around 710 nm.^45^ With this spectral change, the color of the films changed from blue to transparent; that is, π−π* transition at 590 nm decreases and charge carrier bands form between 710 and 1100 nm (Figure S6). The PEDOT:PSS/ITO electrode_2 has also led to a more intense charge carrier band in the NIR region. This phenomenon is attributed to the porous morphology of PEDOT:PSS/ITO electrode_2 and its consequent low *R*_ct_. The oxidized form of the PEDOT−PSS film shows complete depletion of the π−π* transition and an increase in charge carrier absorption at longer wavelengths. This feature can be attributed to the induced radical cation (polaron) states of the conjugated PEDOT main chain.

**Figure 5:**
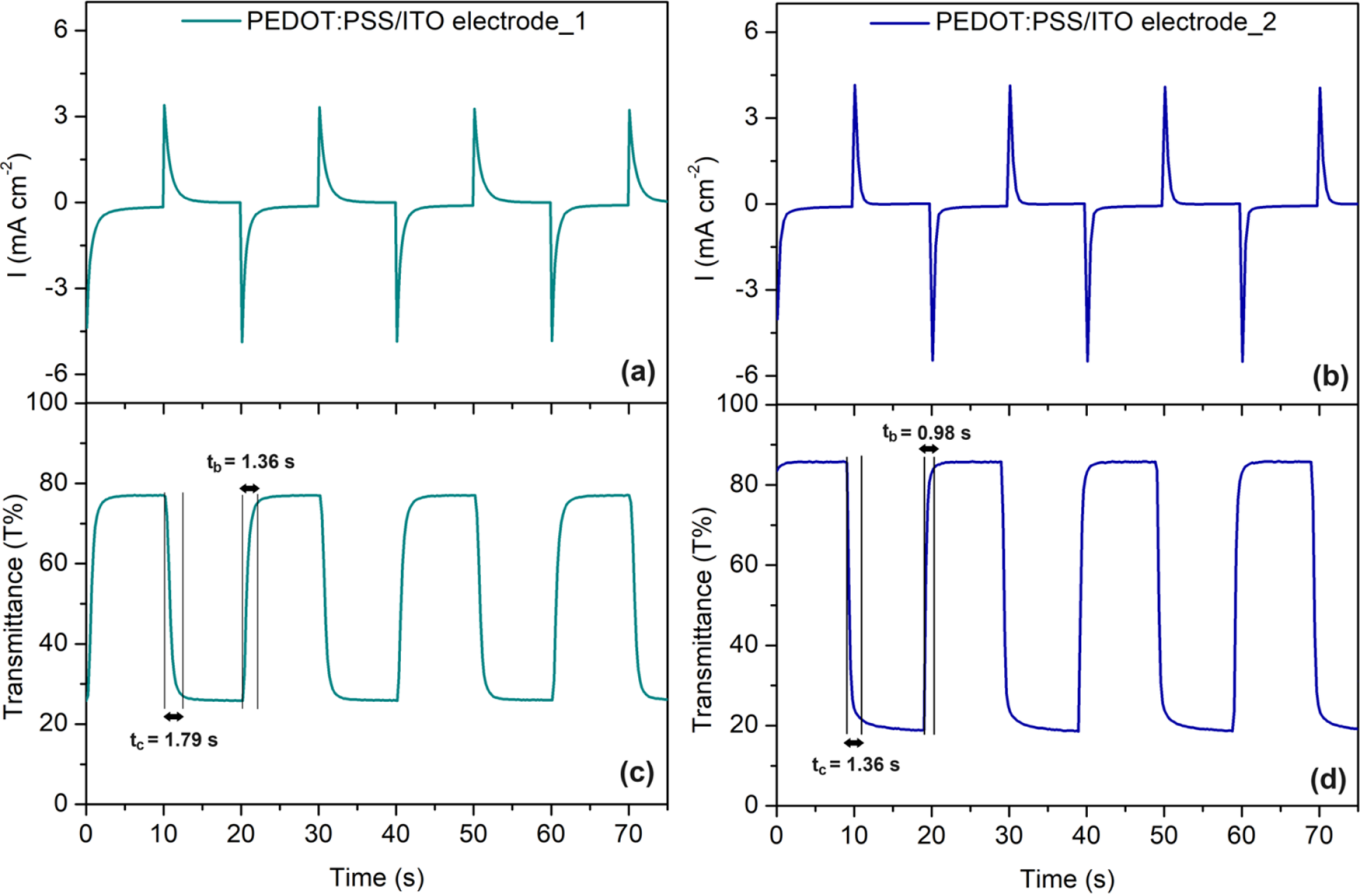
Chronoamperometry curves of PEDOT:PSS/ITO electrode_1 (a) and PEDOT:PSS/ITO electrode_2 (b). The corresponding in situ transmittance curve at 590 nm is shown for PEDOT:PSS/ITO electrode_1 (c) and PEDOT:PSS/ITO electrode_2 (d).

To promote the electrochromic properties of PEDOT:PSS-based electrodes, optical transmittance spectra were recorded at 10 s intervals ranging from −0.9 to 1.0 V. Initially, both PEDOT:PSS/ITO electrode_1 and PEDOT:PSS/ITO electrode_2 exhibited a dark blue color with optical transmittance of approximately 24 and 18% at a wavelength of 590 nm (at −0.9 V), respectively. Upon application of a voltage of 1.0 V to the electrodes, their color converted to transparent, resulting in optical transmittance values of approximately 78 and 84% at the same wavelength, respectively. Therefore, the optical contrast in the visible region between oxidation and reduction increased from 54% in the film fabricated via the spray method to 66% in the film fabricated via the electrospray method. Moreover, in the context of electrochromic materials, the coloration switching time is defined as the duration necessary for an electrode to achieve 90% of the complete transmittance modulation between its stable colored and bleached states.^46^ Chronoamperometry was conducted on the electrodes within a potential range of −0.9 to 1.0 V relative to a Ag wire electrode (Figure 5a,b), and the corresponding in situ transmittance curve was obtained (Figure 5c,d). Upon examination of the transmittance spectrum, the coloring time (*t*_c_) was found to be 1.79 s, while the corresponding bleaching time (*t*_b_) was determined as 1.36 s. These values were enhanced compared to the *t*_c_ of 1.36 s and the corresponding *t*_b_ of 0.98 s observed in the electrode produced via the electrospray method. The rapid switching of PEDOT:PSS/ITO electrode_2 might stem from an enhanced interaction at the interface with the electrolyte during oxidation and reduction, facilitated by the porous structure of the film. Another crucial parameter for electrochromic materials, coloration efficiency (CE) holds as much significance as the time it takes for color change; CE is described as the alteration in optical density per unit of charge injected or ejected at a certain wavelength.^29,47^ The calculated CE value for PEDOT:PSS/ITO electrode_2 is 84.32 cm^2^ C^−1^, which is approximately double the value of PEDOT:PSS/ITO electrode_1 (44.82 cm^2^ C^−1^). Additionally, PEDOT:PSS/ITO electrode_2 demonstrates enhanced cyclic stability compared to that of PEDOT:PSS/ITO electrode_1. After 2000 cycles, retaining 78.8% of the contrast for PEDOT:PSS/ITO electrode_2 represents a significant improvement over PEDOT:PSS/ITO electrode_1, which retained only 55.6% of the contrast (Figure S7). Compared with the literature, the transmittance change and also coloration efficiency are quite acceptable.^28,48−50^

## 4 Conclusions

In summary, this study presents a comparative analysis of the EC-SC performance of electrodes obtained by depositing pristine PEDOT:PSS onto ITO surfaces by using two different methodologies. The findings indicate that films prepared via the electrospray method, known for its simplicity and innovation, exhibit significantly enhanced EC-SC properties compared with those prepared via the spray method. The capacitance values of the prepared electrodes were measured at 826.14 and 1678.60 μF cm^−2^ for PEDOT:PSS/ITO electrode_1 and PEDOT:PSS/ITO electrode_2, respectively, at a current density of 10 μA cm^−2^. Furthermore, PEDOT:PSS/ITO electrode_2 demonstrates a high coloration efficiency (84.32 cm^2^ C^−1^) and rapid response speed (1.36 s for coloration and 0.98 s for bleaching). Notably, this study likely represents the first comparison of the performance of pristine PEDOT:PSS-based electrodes prepared by using two different methods in electrochromic supercapacitors. The innovative and straightforward electrospray approach underscores the potential of these electrodes in multifunctional energy storage devices. In the next stage, these PEDOT:PSS-based electrodes can be paired with an appropriate cathodic layer and an efficient electrolyte that facilitates charge transport for a practical solid-state device.

## Supporting Information Available

Fabrication of the PEDOT:PSS/ITO electrode, characterizations, and calculations (PDF)

## Abbreviations

EC-SC: electrochromic supercapacitor
PEDOT: Poly(3,4-ethylenedioxythiophene)
HTM: hole-transporting material
GCD: galvanostatic charge−discharge
*R*_ct_: charge-transfer resistance
EDL: electrical double layer
*t*_c_: coloring time
*t*_b_: bleaching time
CE: coloration efficiency
