# Supplementary material for: Comparative Study of Electrochromic Supercapacitor Electrodes Based on PEDOT:PSS/ITO Fabricated via Spray and Electrospray Methods
Source: ACS Omega. 2024 Jun 28;9(29):32107–15. doi: 10.1021/acsomega.4c04235 (PMC11270695; doi:10.1021/acsomega.4c04235)
Supplement: Supplementary file 1 — ao4c04235_si_001.pdf [file ao4c04235_si_001.pdf]

Electronic Supporting Information For

Comparative Study of Electrochromic Supercapacitor Electrodes Based on PEDOT:PSS/ITO Fabricated via Spray and Electrospray Methods

Fahri Çatoğlu,[†,ς] Sinem Altınışık[†,ς] and Sermet Koyuncu*[†,ς]*

[†] Department of Chemical Engineering, Canakkale Onsekiz Mart University, 17100 Canakkale, Türkiye.

[ς] Department of Energy Resources and Management, Canakkale Onsekiz Mart University, 17100 Canakkale, Türkiye.

sinemaltinisik@comu.edu.tr ; skoyuncu@comu.edu.tr

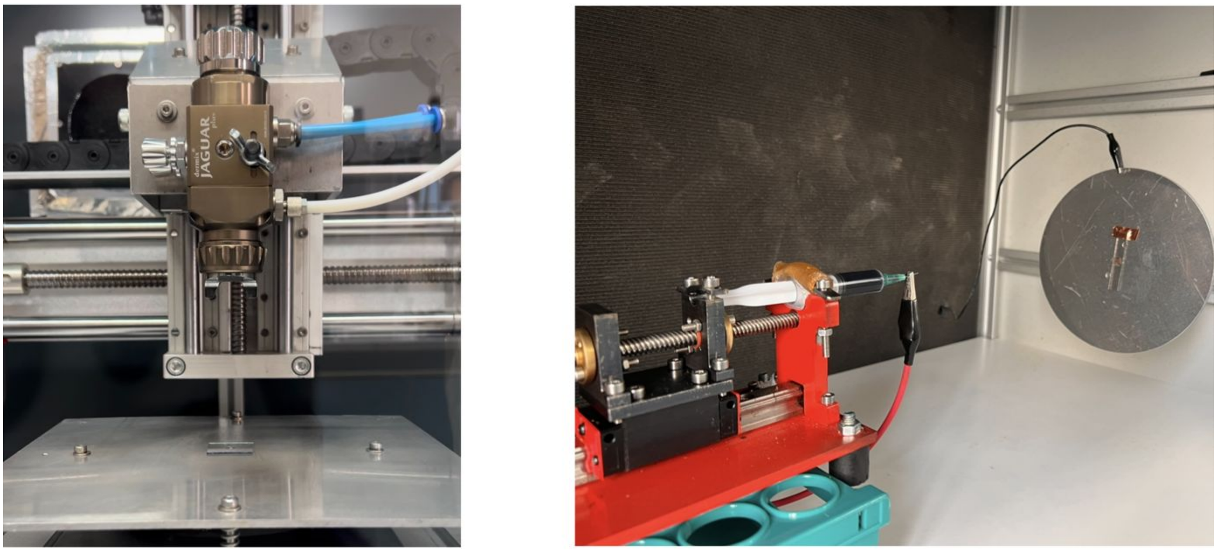

(a) (b)

Figure S1. Spray (a) and electrospray (b) coating devices.

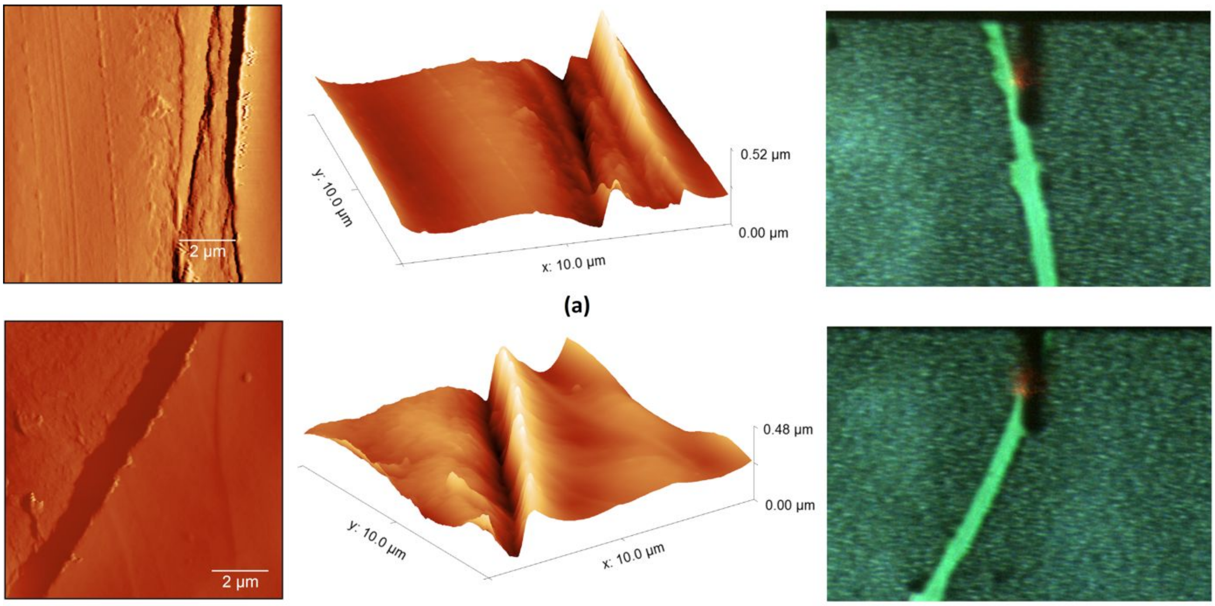

(b)

Figure S2. AFM and microscope images of (a) PEDOT:PSS/ITO electrode_1 and (b) PEDOT:PSS/ITO electrode_2.

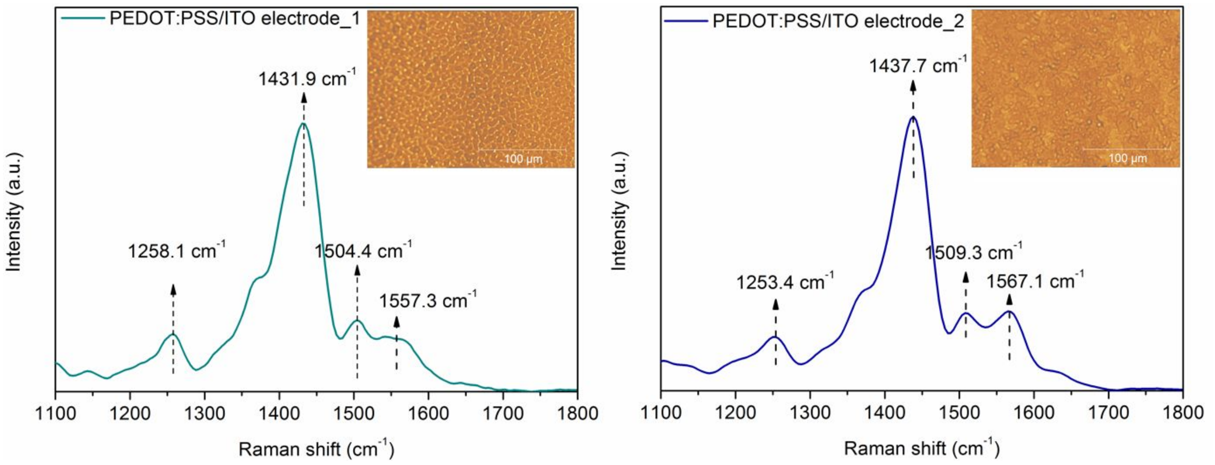

Figure S3. Raman spectra of PEDOT:PSS/ITO electrode_1 and PEDOT:PSS/ITO electrode_2.

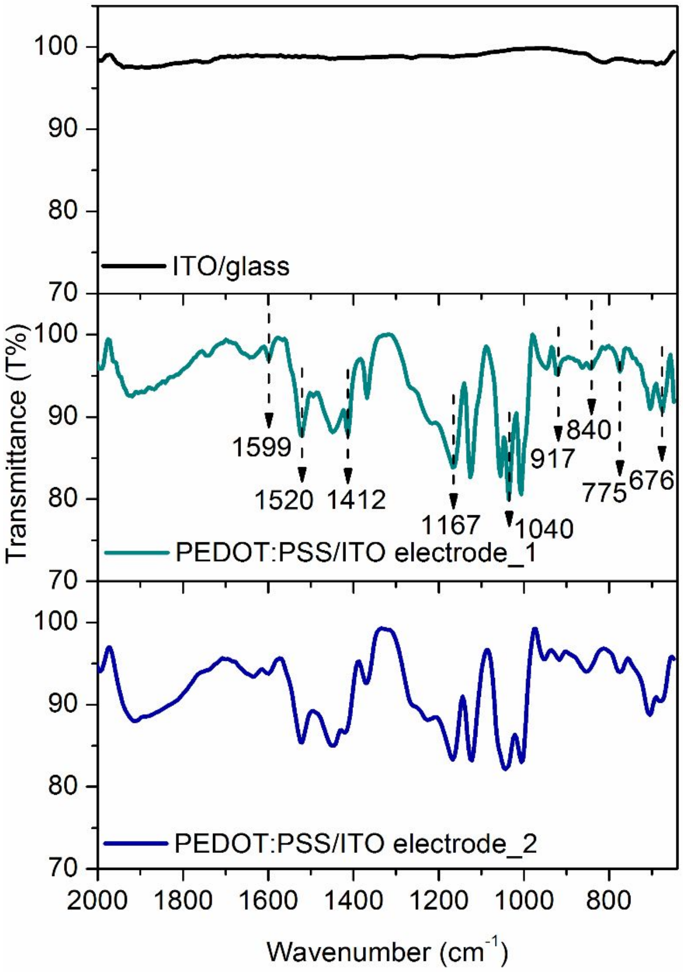

Figure S4. FTIR spectra of ITO/glass, PEDOT:PSS/ITO electrode_1 and PEDOT:PSS/ITO electrode_2.

Calculations

Coloration efficiency was calculated using the following equation:

$$\log(T_b(\lambda) / T_c(\lambda)) = \Delta\text{optical density} \quad (1)$$

$$\Delta\text{optical density} / Q_d = CE \quad (2)$$

where Q_d refers to the injected/ejected charge during the redox process, and T_b and T_c refer to the transmittances of the material in its bleached and colored states, respectively.

$$I\ \Delta t / \Delta V\ S = C \quad (3)$$

where C stands for the areal capacitance (F cm^{-2}), S represents the surface area of the active materials (cm^2), V is the potential window (V), I is the discharge current (mA), and t stands for the discharge time (s).

$$I(V) = k_1\nu + k_2\nu^{0.5} \quad (4)$$

$$I(V)/\nu^{0.5} = k_1\nu^{0.5} + k_2 \quad (5)$$

where *i* stands for the current (mA), ν represents the sweep rate (V s^{-1}), $k_1\nu$ and $k_2\nu^{0.5}$ correspond to the current contributions from the surface capacitive effects and the diffusion-controlled intercalation process, respectively. Fig. S2 plots the sweep rate dependence of the current according to eq. 5 for constant potentials between 0.0 and 0.9 V.

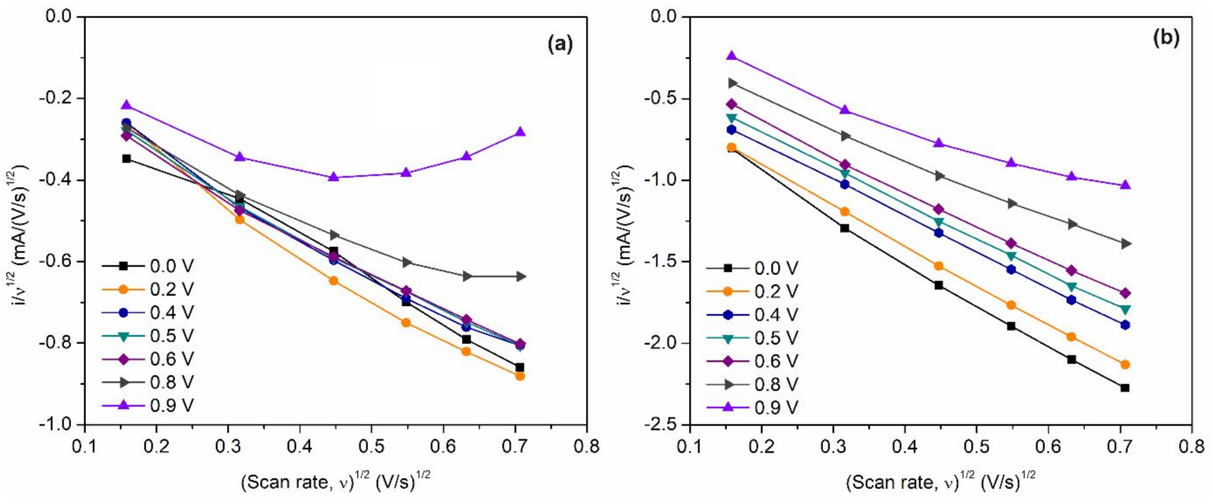

Figure S5. Use of Dunn equation to analyze the cathodic voltametric sweep data for PEDOT:PSS/ITO electrode_1 (a) and PEDOT:PSS/ITO electrode_2 (b). Sweep rates were varied from 25 to 50 mV/s.

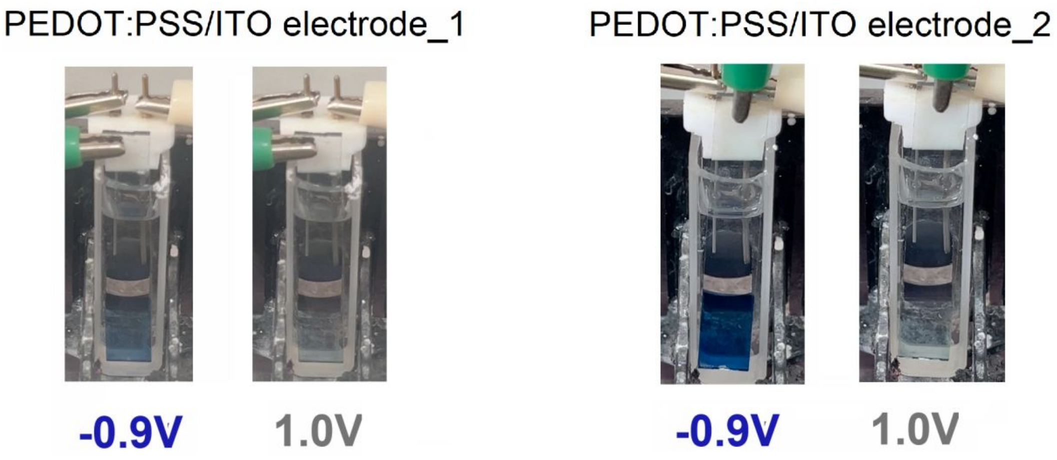

Figure S6. Change of color of electrodes from blue to transparent.

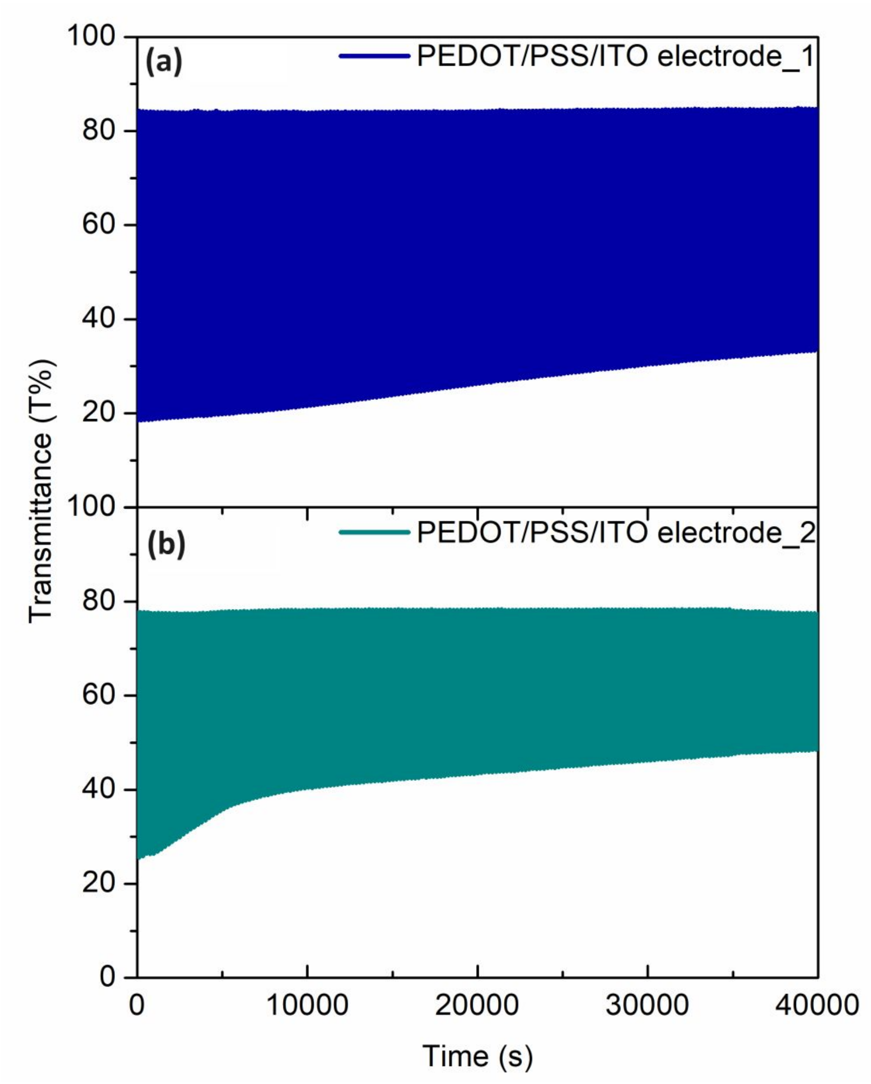

Figure S7. Transmittance-time profiles of PEDOT:PSS/ITO electrode_1 (a) and PEDOT:PSS/ITO electrode_2 (b) after 2000 cycles from -0.9 to 1.0 V.

Tablo S1. Comparison of EC-SC performances and properties of PEDOT:PSS/ITO based electrodes with previous studies.

Electrode material	Electrolyte	Voltage window	Capacitance	Energy density	Power density	T%	Ref
PEDOT:PSS/Ag grids	PVA-H_3PO_4 gel	0.0 V - 0.8 V	7.36 mF cm^{-2}	0.59 mW h cm^{-2}	0.036 W cm^{-2}		[1]
DMSO doped PEDOT:PSS/ Cellulose/ polyester cloth	Sweat (artificial and human)	0.0 V - 1.4 V	8.94 F g^{-1} (10 mF cm^{-2})	1.63 µWh cm^{-2}	0.40 mW cm^{-2}		[2]
PEDOT:PSS/flexible plastic substrates (PET)	0.5 M K_2SO_4 (aq)	0.1 - 0.7 V	1.0 mF cm^{-2}			81	[3]
PEDOT:PSS fiber	PVA-H_3PO_4 gel	0.0 - 1.1 V	119 mF cm^{-2}	4.13 µWh cm^{-2}	250 µW.cm^{-2}		[4]
PEDOT: PSS/AgNFs	PVA-H_2SO_4 gel	0.0 - 1.0 V	3.64 mF cm^{-2}			~85	[5]
PEDOT:PSS/AgNWs	PVA-H_3PO_4 gel	0.0 V - 0.8 V	0.6 mF cm^{-2}			~51	[6]
Embedded Ni mesh/PEDOT:PSS	PVA-H_3PO_4 gel	-0.6 – 0.6	0.52 mF cm^{-2}			83	[7]
PEDOT:PSS/PET	PVA-H_3PO_4 gel	0.0 V - 0.8 V	1.18 mF cm^{-2}	0.38 mW h cm^{-2}	0.036 W cm^{-2}		[8]
RuO_2/PEDOT:PSS	PVA-H_2SO_4 gel	0.0 -1.2 V	1.2 mF cm^{-2}	0.053 µWh cm^{-2}	147 µW.cm^{-2}		[9]
PEDOT-coated polyester fabric	1 M Na_2SO_4 solution	-0.2 – 0.8 V	0.64 F cm^{-2}				[10]
WO_3 nanoparticles on the silver grid/PEDOT:PSS hybrid film	0.5 M H_2SO_4	-0.7 – 0.0	221 F g^{-1}			81.9	[11]
PEDOT:PSS/ Carbon yarns	Cellulose acetate (CA) gel-polymer electrolyte	0.0-1.0 V	72 mF g^{-1}				[12]
MWCNT/cellulose/ PEDOT:PSS	PVA/KOH	0.0-1.0 V	380 F g^{-1} @0.25 A g^{-1}	13.2 Wh kg^{-1}	0.126 kW kg^{-1}		[13]
PEDOT:PSS/silver nanowires	PVA/H_3PO_4	0.0-1.0 V	113 F g^{-1} @10 mV s^{-1}			87	[14]
**PEDOT:PSS/ITO electrode_1;** **PEDOT:PSS/ITO electrode_2**			**826.14 µF cm^{-2}** for electrode_1; **1678.60 µF cm^{-2}** for electrode_2	**0.41 mW h cm^{-2}** for electrode_1 **0.84 mW h cm^{-2}** for electrode_2	**4.96 µW cm^{-2}** for electrode_1 **4.97 µW cm^{-2}** for electrode_2		**This work**

REFERENCES

(1) Cheng, T.; Zhang, Y. Z.; Yi, J. P.; Yang, L.; Zhang, J. D.; Lai, W. Y.; Huang, W. Inkjet-printed flexible, transparent and aesthetic energy storage devices based on PEDOT: PSS/Ag grid electrodes. *J. Mater. Chem. A* **2016**, 4(36), 13754-13763.

(2) Manjakkal, L.; Pullanchiyodan, A.; Yogeswaran, N.; Hosseini, E. S.; Dahiya, R. A wearable supercapacitor based on conductive PEDOT: PSS-coated cloth and a sweat electrolyte. *Adv. Mater.* **2020**, 32(24), 1907254.

(3) Higgins, T. M.; Coleman, J. N. Avoiding resistance limitations in high-performance transparent supercapacitor electrodes based on large-area, high-conductivity PEDOT: PSS films. *ACS Appl. Mater. Interfaces.* **2015**, *7*(30), 16495-16506.

(4) Yuan, D.; Li, B.; Cheng, J.; Guan, Q.; Wang, Z.; Ni, W.; Li, C.; Liu, H.; Wang, B. Twisted yarns for fiber-shaped supercapacitors based on wetspun PEDOT: PSS fibers from aqueous coagulation. *J. Mater. Chem. A* **2016**, 4(30), 11616-11624.

(5) Singh, S. B.; Kshetri, T.; Singh, T. I.; Kim, N. H.; Lee, J. H. Embedded PEDOT: PSS/AgNFs network flexible transparent electrode for solid-state supercapacitor. *J. Chem. Eng.* **2019**, 359, 197-207.

(6) Liu, X.; Li, D.; Chen, X.; Lai, W. Y.; Huang, W. Highly transparent and flexible all-solid-state supercapacitors based on ultralong silver nanowire conductive networks. *ACS Appl. Mater. Interfaces.* **2018**, 10(38), 32536-32542.

(7) Liu, Y. H., Xu, J. L.; Shen, S.; Cai, X. L.; Chen, L. S.; Wang, S. D. High-performance, ultra-flexible and transparent embedded metallic mesh electrodes by selective electrodeposition for all-solid-state supercapacitor applications. *J. Mater. Chem. A* **2017**, 5(19), 9032-9041.

(8) Cheng, T.; Zhang, Y. Z.; Zhang, J. D.; Lai, W. Y.; Huang, W. High-performance free-standing PEDOT: PSS electrodes for flexible and transparent all-solid-state supercapacitors. *J. Mater. Chem. A* **2016**, 4(27), 10493-10499.

(9) Zhang, C. J.; Higgins, T. M.; Park, S. H.; O'Brien, S. E.; Long, D.; Coleman, J. N.; Nicolosi, V. Highly flexible and transparent solid-state supercapacitors based on RuO2/PEDOT: PSS conductive ultrathin films. *Nano Energy* **2016**, 28, 495-505.

(10) Yu, X.; Su, X.; Yan, K.; Hu, H.; Peng, M.; Cai, X.; Zou, D. Stretchable, conductive, and stable PEDOT-modified textiles through a novel in situ polymerization process for stretchable supercapacitors. *Adv. Mater. Technol.* **2016**, 1(2), 1600009.

(11) Cai, G.; Darmawan, P.; Cui, M.; Wang, J.; Chen, J.; Magdassi, S.; Lee, P. S. Highly stable transparent conductive silver grid/PEDOT: PSS electrodes for integrated bifunctional flexible electrochromic supercapacitors. *Adv. Energy Mater*. **2016**, 6(4), 1501882.

(12) Moniz, M. P.; Rafique, A.; Carmo, J.; Oliveira, J. P.; Marques, A.; Ferreira, I. M.; Baptista, A. C. Electrospray Deposition of PEDOT: PSS on Carbon Yarn Electrodes for Solid-State Flexible Supercapacitors. *ACS Appl. Mater. Interfaces* **2023**, 15(25), 30727-30741.

(13) Zhao, D.; Zhang, Q.; Chen, W.; Yi, X.; Liu, S.; Wang, Q.; Liu, Y.; Li, J.; Li, X.; Yu, H. Highly flexible and conductive cellulose-mediated PEDOT: PSS/MWCNT composite films for supercapacitor electrodes. *ACS Appl. Mater. Interfaces* **2017**, 9(15), 13213-13222.

(14) Zhu, W. C.; He, P. Q.; Tien, H. C.; Liu, H. L.; Chen, W. C.; Lv, W.; Lee, W. Y. Solvent-enhanced transparent stretchable polymer nanocomposite electrode for supercapacitors. *ACS Appl. Energy Mater.* **2021**, 4(3), 2266-2274.
